# Microwave Assisted Preparation of Antimicrobial Chitosan with Guanidine Oligomers and Its Application in Hygiene Paper Products

**DOI:** 10.3390/polym9120633

**Published:** 2017-11-24

**Authors:** Junrong Li, Ying Ye, Huining Xiao, Beihai He, Liying Qian

**Affiliations:** 1 State Key Laboratory of Pulp and Paper Engineering, South China University of Technology, Guangzhou 510640, China; lljrr@scut.edu.cn (J.L.); yeyingshen@126.com (Y.Y.); ppebhhe@scut.edu.cn (B.H.); 2 Department of Chemical Engineering, University of New Brunswick, Fredericton, NB E3B 5A3, Canada; hxiao@unb.ca

**Keywords:** chitosan, guanidine, microwave irradiation, antimicrobial activity, hygiene paper

## Abstract

Guanidinylated chitosan (GCS) was prepared by grafting guanidine oligomers onto chitosan under microwave irradiation. The structure of GCS characterized by FT-IR and ^1^H NMR verified the covalent bonding between the guanidine oligomers and chitosan; the effects of molar ratio, reaction temperature, and time were investigated and the degree of substitution of GCS reached a maximum of 25.5% under optimized conditions in this work. The resulting GCS showed significantly enhanced antimicrobial activities. The results obtained from the dynamic UV absorption of *Escherichia coli* (*E. coli*) and atomic force microscopy (AFM) revealed that the deactivation of *E. coli* by GCS was due to the destructing of the cell membrane and the prompt release of cytoplasm from the bacterial cells. The adsorption of GCS onto cellulose fibers and the antimicrobial efficiency of the hygiene papers with GCS were also investigated. Microwave irradiation as a green assisted method was applied to promote this reaction. This facile approach allowed chitosan to be guanidinylated without tedious preparation procedures and thus broadened its application as a biocompatible antimicrobial agent.

## 1. Introduction

Chitosan is one of the natural macromolecules which attracts extensive interest due to non-toxicity, biodegradability, biocompatibility, antimicrobial and antifungal activities. The primary amino groups present in the molecular chains impart positive charges to chitosan in acidic solutions [1], therefore, chitosan can inhibit a broad spectrum of bacteria by electrostatic interaction between the primary amino groups of chitosan and the phosphoryl groups of phospholipid components of cell membranes [2]. The unique properties of chitosan give it important applications such as in tissue engineering [3,4,5,6,7,8,9]. However, the insolubility in aqueous solution and lower antimicrobial activity than chemical biocides restrict its further application and development. Chitosan has three reactive groups, i.e., amino groups (C–2), as well as primary (C–6) and secondary (C–3) hydroxyl groups which are readily subject to chemical modifications. In order to enhance the antimicrobial activity of chitosan, some more positively charged groups such as quaternary amine [10,11,12] and guanidine [13,14,15] have been endowed onto chitosan chains. Grafting those groups onto chitosan normally requires a reaction conducted at an elevated temperature. One of the recently developed approaches for heating the reaction system is microwave assisted heating which appears to be more homogeneous, selective and efficient in comparison with the conventional heating method [16,17,18]. The microwave irradiation heating process also resulted in fast reaction with few or no side products. The effect of microwave irradiation in chemical reactions is a combination of thermal effect and non-thermal effects, in addition to effects on mobility and diffusion that may increase the probabilities of effective contacts [19]. The design of various types of macromolecular architectures with control of structural parameters have been emphasized in order to achieve strong antimicrobial activities with targeting inhibition while minimizing toxicity to mammalian cells [20,21]. Cationic compounds such as surfactants, lipids, peptides and polymers are promising candidates for development of antimicrobial agents [22]. A guanidine-based polymer is a series of cationic polyelectrolytes bearing guanidino groups, which can be obtained by grafting guanidine salt [23], guanidine oligomers [24,25] and arginine [26,27] to the polymers, such as β-CD [23], PAN [25], PVC [24], PSI [27], chitosan [26], cellulose [28], and by polymerization with guanidinylated monomer [29] or a postpolymerizationguanylation method [30]. The guanidine groups could be fully protonated at physiological pH, leading to cationic guanidinium ions [31] which exhibit a highly effective antimicrobial performance. More guanidine groups can be rendered to the polymers with guanidine oligomers compared to other small molecules. Polyhexamethylene guanidine hydrochloride (PHGC) is one of the guanidine oligomers synthesized by condensation of hexamethylene diamine and guanidine hydrochloride at elevated temperature, which has been used extensively and safely as disinfectant and biocide due to its extremely outstanding antibacterial behavior (minimum inhibition concentration (MIC) ≤ 10 ppm) [32,33]. Meanwhile the primary amine groups in both chitosan and PHGC are highly reactive so that PHGC has mainly potential to be grafted onto chitosan by condensation without crosslinking agents. Works on guanidinylating modification of chitosan to enhance its antibacterial activity have been reported recently [14,15,26,31,34,35,36,37,38,39,40]. However, only one or two guanidine groups were born in each pendent chain of the obtained guanidinylated chitosan (GCS) by applying crosslinking agents or multiple reaction procedures. Chitosan/polymeric guanidine complexes were also prepared to obtain synergistic effects of enhancing antimicrobial activity and wet-strength of paper; however, the guanidine polymers included in the complexes were released rapidly, thus jeopardizing the antimicrobial performance [41]. To our knowledge, GCS with guanidine oligomers has not been reported so far, especially obtained by the facial method of microwave irradiation. Moreover, GCS is a good additive potentially for hygiene paper such as banknotes, medical paper, tissues and food wrappers because of its non-toxicity, biodegradability and antimicrobial activity. GCS bearing more cationic guanidinium ions is likely to be adsorbed on the cellulose fibers and retained in the paper due to the high molecular weight of chitosan, which avoids utilizing PHGC with carboxymethylcellulose as polyelectrolyte complex [42].

In this work, the grafting of guanidine oligomers (PHGC) to chitosan was conducted via a microwave assisted heating process, which provided a feasible method to obtain GCS with enhanced antimicrobial activity. The molecular structure of GCS was characterized and various influencing factors, including microwave irradiation conditions, were investigated in an attempt to optimize the conditions for the reaction. The antimicrobial activity of chitosan was significantly enhanced by rendering the guanidine groups, and *E. coli* was deactivated by releasing the cytoplasm from the destructive cells. The cationic GCS could be adsorbed onto the cellulose fibers and then be applied to make hygiene papers. The antimicrobial efficiency of GCS addition amounts and methods were also investigated.

## 2. Materials and Methods

### 2.1. Materials

Chitosan (deacetylation degree = 80%; 200–400 KDa) purchased from Aladdin (Shanghai, China) was further deacetylated to increase the deacetylation degree to 95%, determined by an elemental analyzer (Vario EL III, Elementar Analysensysteme GmbH, Langenselbold, Germany). Hexamethylene diamine, guanidine hydrochloride and other chemicals (Aladdin, Shanghai, China) were used without further purification. *E. coli* (ATCC11229), LB broth, LB agar and PBS were supplied from Huankai Microbial Sci. & Tech. Co. Ltd. (Guangzhou, China). Eucalyptus fibers were obtained from YueYang Paper Co. Ltd. (Yueyang, China) which were beaten to 30 °SR by PFI (V1, Norwegian Pulp and Paper Research Institute, Oslo, Norway) at a high consistency (10 wt %). PHGC was prepared by condensation polymerization of hexamethylene diamine and guanidine hydrochloride.

### 2.2. Preparation of GCS

The deacetylated chitosan (1% *w/v*) was dissolved in 0.15 mol L^−1^ HCl and a certain amount of PHGC aqueous solution (40 wt %) was also added into the flask. The mixture was irradiated at 700 watts in a microwave reactor (MicroSYNTH, Milestone, Milan, Italy). The resulting products were washed with ethanol twice, dialyzed with a dialysis tube with a molecular weight cut off of 10,000 in deionized water to remove the unreacted PHGC, and lyophilized to obtain the GCS.

### 2.3. Characterization of GCS

FT-IR spectra of chitosan and GCS were recorded using the KBr disk method (Bruker Vector 33, Bruker Corporation, Karlsruhe, Germany). ^1^H NMR spectra were recorded on a Bruker Avance 400 NMR Spectrophotometer (Bruker Corporation, Karlsruhe, Germany) using 1 wt % CD_3_COOD in D_2_O as solvent to characterize the structures of chitosan and GCS. All samples were further vacuum dried at 60 °C for 2 days prior to the measurements. The degree of substitution (*DS*) of GCS was calculated by the followed equations where *C*/*N* was determined by elemental analysis (Vario EL III Elemental Analyzer, Elementar Analysensysteme GmbH, Langenselbold, Germany), *m* is the polymerization degree of PHGC, and 95% is the deacetylation degree of chitosan.

(1) $$C/N=\frac{6\times 12\times 95\%+7\times 12\times 5\%+7\times 12\times m\times DS}{1\times 14+3\times 14\times m\times DS}=\frac{72.6+84\times m\times DS}{14+42\times m\times DS}$$

(2) $$DS=\frac{14C/N-72.6}{(84-42C/N)\times m}$$

### 2.4. Antimicrobial Activity of GCS

A serial dilution method was used to determine the MIC of chitosan and GCS against *E. coli*. Dynamic UV absorption of *E. coli* suspension untreated or treated with GCS was measured using a UV-Vis spectrophotometer (Hach DR5000, Hach Company, Loveland, CO, USA). AFM images of the bacteria were obtained in tapping mode using a silica probe on a Nanoscope IIIA (Veeco Probes Nanofabricaiton Center, Camarillo, CA, USA).

### 2.5. Adsorption of GCS on Cellulose Fibers

Bleached sulfate pulp of Eucalyptus (1 g) was dispersed in DD water in a flask and GCS solution was added to the flask to make atotal weight of pulp slurry of 50 g, meanwhile the pH was adjusted to 6 with acetic acid. The flask was shaken for 30 min at 200 rpm and 30 °C, then the slurry was vacuum filtered and the adsorbed amount of GCS was quantified by a colloidal titration with Potassium Polyvinyl Sulfate (PVSK) solution (0.5 mN) (Mütek PCD04, BTG Instruments GmbH, Wessling, Germany). The amount of adsorption of cationic polymer (mg·g^−1^) based on the weight of cellulose fibers is calculated as *Γ*_w_ = (*V*_1_ − *V*_2_)∗*C/V*_1_, where *V*_1_ and *V*_2_ are the volume (mL) of PVSK to titrate control and sample, respectively. *C* is the concentration of GCS (mg·g^−1^ o.d. (oven dry) pulp).

### 2.6. Hygiene Paper and Its Inhibition Efficiency

Hygiene paper was prepared by two approaches, i.e., adding GCS as additive or spaying GCS on the surface of handsheets. Handsheets of 60 g·m^−2^ were prepared in a standard format with/without defined amounts of GCS. For saturated samples, fibers were stirred with GCS for 30 min before making handsheets. For sprayed samples, GCS solution was sprayed directly onto the surface of the handsheets. The inhibition efficiency of the hygiene paper was determined by the shaking flash method. A quantity of 0.75 g of paper scraps was dispersed into a suspension of 5 mL of bacterial (10^6^ CFU·mL^−1^) and 70 mL PBS, shaking at 200 rpm at 37 °C for 1 h. Then, 0.5 mL of this culture was seeded on an agar plate. The plates were incubated at 37 °C for 24 h and the number of colonies was counted (three repeats for each sample). Inhibition efficiency (%) is related to the deactivated bacteria compared with the control.

## 3. Results

### 3.1. Synthesis and Characterization of GCS

**Scheme 1:**
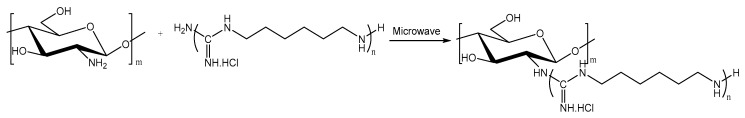
The synthesis process of guanidinylated chitosan under microwave irradiation.

**Figure 1:**
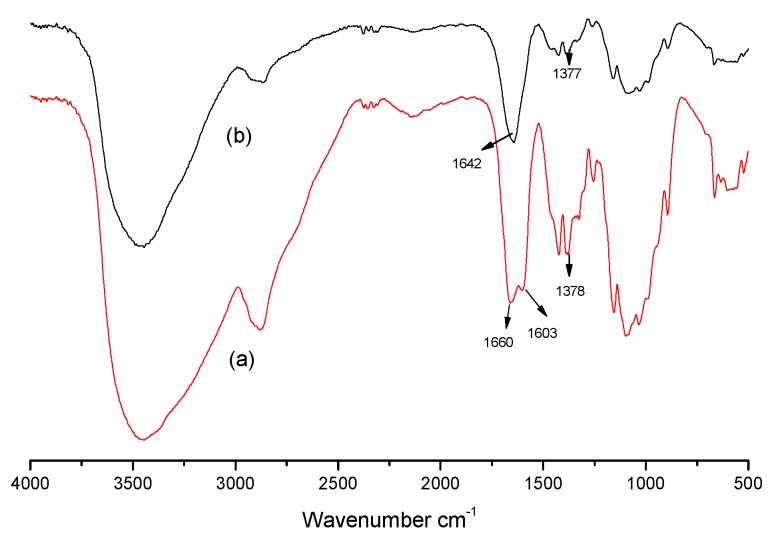
FT-IR spectra of chitosan (**a**), and guanidinylated chitosan (GCS) (**b**).

In this work, GCS was synthesized by grafting guanidine oligomers onto the molecular chain of chitosan under microwave irradiation with the intention of enhancing antimicrobial activity. Scheme 1 presents the reaction of chitosan with PHGC. The two amino groups from chitosan and PHGC were condensed by the release of ammonia gas. FT-IR and ^1^H NMR were applied to characterize the structure of GCS. Comparing FT-IR spectra of chitosan with GCS (Figure 1), the peak at 1603 cm^−1^ was assigned to the bending vibration of the –NH_2_ group in chitosan and disappeared in the spectrum of GCS which indicated that the primary amine groups in chitosan were consumed as PHGC was grafted onto it. Meanwhile, a new peak at 1642 cm^−1^ emerged which was assigned to the stretching vibration of C=NH in the guanidine group of PHGC [43]. The peak at 1660 cm^−1^ of chitosan corresponds to the C=O stretching vibration in the –NHCOCH_3_ group and it almost disappeared after the reaction of chitosan and PHGC.

**Figure 2:**
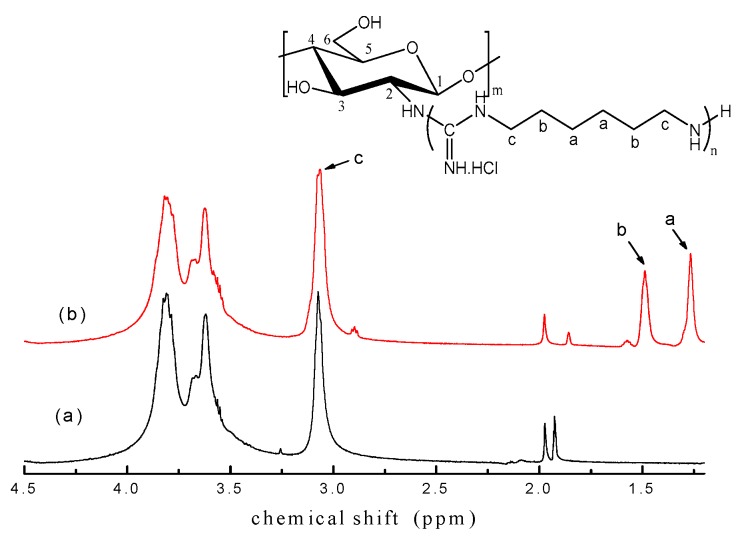
^1^H NMR spectra of chitosan (**a**) and GCS (**b**) (Temperature = 90 °C; Molar ratio = 2.5:1; 30 min).

^1^H NMR spectra of chitosan and GCS are shown in Figure 2. For ^1^H NMR spectrum of chitosan, typical signals of the saccharide proton peaks at 3.06 ppm (H–2), 3.61–3.70 ppm (H–5, 6), 3.82 ppm (H–3, 4), 1.85 ppm (HN–COCH_3_) and residual protons of the solvent at 1.96 ppm (CH_3_COOD) were detected [17,44]. ^1^H NMR spectrum of GCS was similar to that of chitosan except for new peaks appearing at 1.27 ppm (H–a), 1.49 ppm (H–b) which were assigned to the protons on the PHGC [45]. The chemical shift of methylene H–c was assigned to 3.06 ppm as well because the chemical environment of the methylene H–c was very similar to the H–2 of chitosan, which was further downfield by directly connecting with the electronegative amino group. Besides, the signal at 3.06 ppm became relatively strong, compared with the peaks at 3.70 and 3.82 ppm due to the overlapping of the H–2 of chitosan with H–c of PHGC. It was concluded that the guanidine oligomers were covalently incorporated into chitosan. Meanwhile, the peak at 1.85 ppm assigned to the acetyl proton of chitosan decreased in GCS because of deacetylation. In the reaction of chitosan with PHGC, NH_3_ was released and then dissolved in the water, the resultant (NH_3_·H_2_O) is ionized to NH_4_^+^ and OH– making the pH turn weakly alkaline. Deacetylation of chitosan was normally conducted in alkaline condition and microwave irradiation proved to be a more efficient method to accelerate the deacetylation [46]. Therefore, the acetyl groups removed under microwave in weak alkaline condition in this work resulted in a decrease of the peak intensity at 1.85 ppm. The *DS* of GCS in this condition is 25.5% from the area ratio of the CH_2_ proton (H–b) in the side chain of PHGC and the H–5, 6 proton of the chitosan backbone which corresponds to *DS* (25.7%) obtained from elemental analysis.

### 3.2. Influencing Factors on the Grafting of PHGC to Chitosan

**Figure 3:**
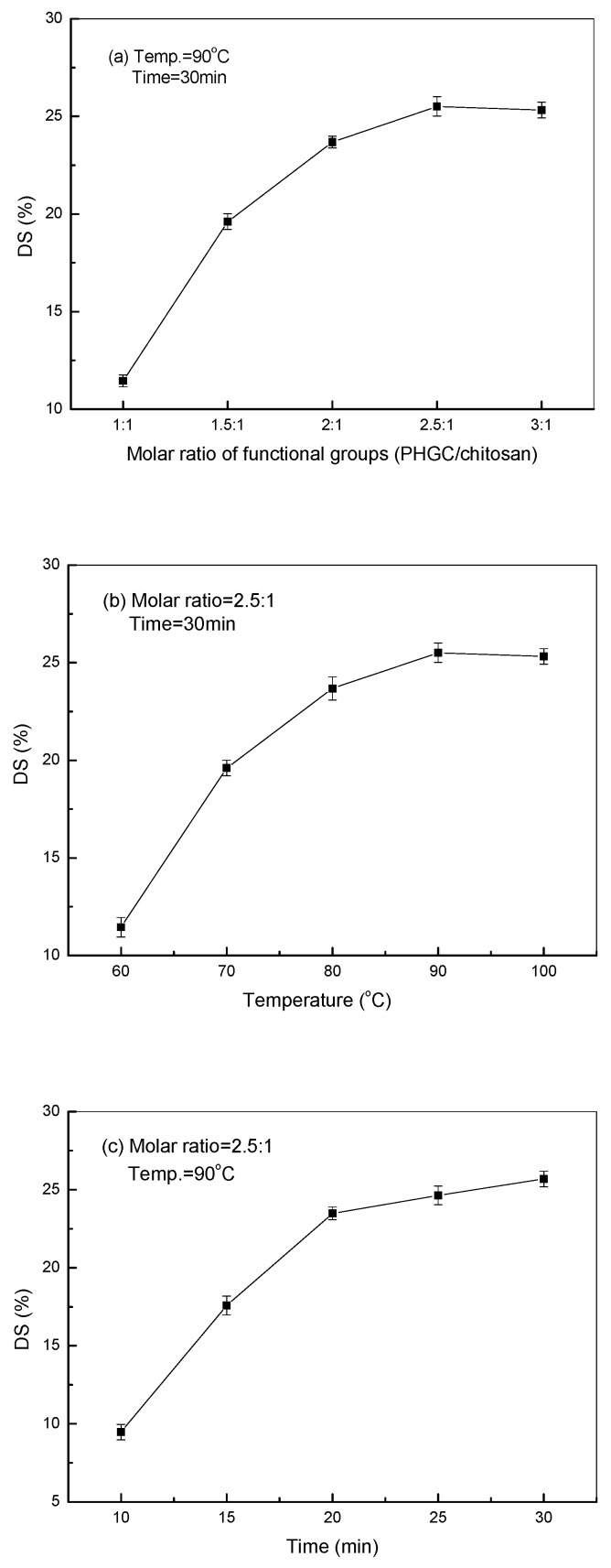
Effects of molar ratio (**a**), microwave temperature (**b**), and time (**c**) on the degree of substitution (*DS*) of GCS.

The molar ratio of the functional groups, the microwave irradiation temperature, and the time are the most important factors influencing the grafting of PHGC to chitosan. The reactions between guanidine oligomers and chitosan were achieved by the effective collisions of the functional groups at a certain temperature and period of time. As shown in Figure 3a, the *DS* increased with a higher molar ratio of functional groups (PHGC/chitosan) from 1:1 to 2.5:1, where the more effective collisions occurred when more guanidine oligomers surrounded the chitosan molecules. However, the *DS* could not be increased further at a higher molar ratio because the grafted guanidine oligomers rendered their positive charges to chitosan which repelled the cationic ungrafted oligomers by electrostatic repulsion. Figure 3b showed that the *DS* of GCS increased from 11.5% to 25.5% when the microwave irradiation temperature was elevated from 60 to 90 °C. More energy at higher temperature can accelerate the thermal motions of molecules and activate the functional groups which here facilitated the condensation between the guanidine oligomers and chitosan. However, the *DS* even decreased when the temperature was up at 100 °C which is the boiling point of the solution. In general, the longer the irradiation time, the higher is the *DS*. The *DS* increased significantly to 23.5% when the period of reaction time was prolonged to 20 min, it was increased only about 1% in 5 min, after that. From Figure 3, it can be concluded that the highest *DS* of GCS (25.5%) was obtained with microwave irradiation assistance in this paper when the molar ratio of the functional groups (PHGC/chitosan) was 2.5:1 at 90 °C and 30 min.

### 3.3. Antimicrobial Activity of GCS

**Figure 4:**
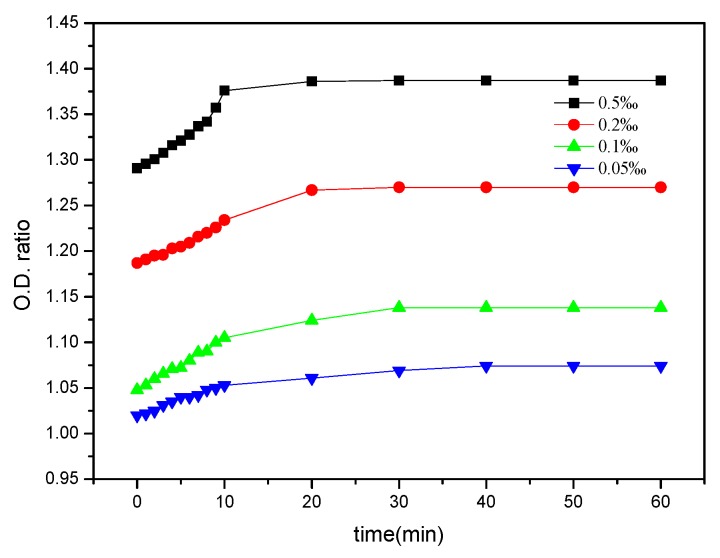
Dynamic UV absorption of *Escherichia coli* (*E. coli*) with various concentrations of GCS (*DS* = 25.5%).

GCS is a kind of cationic polyelectrolyte which can easily attack the anionic surface of bacterial cells through electrostatic attraction, thus leading to breakage of the bacterial cell membrane. Further deacetylation of chitosan and grafting of PHGC can both increase the p*K*a of GCS while more amino groups are protonated to NH^3+^ at pH lower than the p*K*a. Consequently this resulted in the increased antimicrobial activity of GCS to some extent. Moreover, the guanidine groups in the PHGC side chain are the most important because of the significant increase of antimicrobial activity of GCS. Guanidines are the strongest organic bases (p*K*a = 13.5) due to the resonance stabilization of their conjugated acids and the guanidinium cation is very stable in aqueous solution over a wide pH range [47]. Cationic guanidine groups bind to negatively charged cell walls and the membranes of a bacteria, causing its destruction followed by cell fluid leakage, cell growth inhibition and finally death of the bacteria [48]. However, the research of polyguanidinium oxanorbornene revealed that the polymers acted on intercellular targets rather than through a membrane disruption mechanism [49]. Therefore, the molecular structures bearing guanidine groups might influence the antimicrobial mechanism of guanidinylated polymers. As the intracellular components leaking out from cells show a specific absorbance at 260 nm [50], the OD_260_ ratio obtained by UV spectrometry over different time intervals represents the dynamic antimicrobial activity of the GCS against *E. coli*. The higher OD_260_ ratio revealed a higher antibacterial efficiency of GCS. The dynamic UV absorption of *E. coli* treated with GCS at various concentrations is presented in Figure 4. The MIC of PHGC was 7.8 ppm from previous work [51] and the MIC of GCS (*DS* = 25.5%) against *E. coli* ATCC11229 was 0.15‰, which was determined by a serial dilution method, whereas the MIC of chitosan was 1‰. As can be seen, the OD_260_ ratio of *E. coli* increased in the first ten minutes regardless of GCS concentration, and leveled off after 20 min, implying GCS could damage the bacterial cells rapidly and prolonging the time was unlikely to kill more bacteria. When the concentration of GCS was lower than MIC (0.05‰ and 0.1‰), the final OD_260_ ratios were below 1.20. Once the concentration was above MIC, the ratios reached 1.28, or higher, indicating that the membrane of the bacteria was destroyed and more cytoplasm leaked from damaged *E. coli* cells. Guanidine oligomers could damage most of the bacterial cells in a few seconds when the concentration was sufficiently high. The covalent bonding of the guanidine oligomers onto chitosan rendered highly enhanced GCS antimicrobial activity.

**Figure 5:**
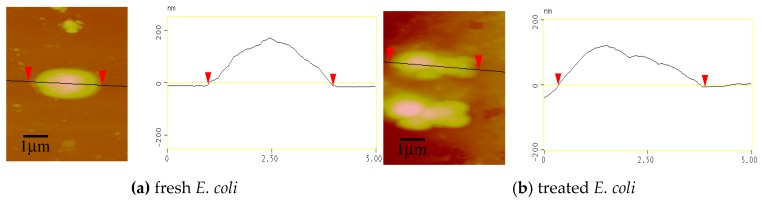
Height and section images of fresh (**a**) and treated *E. coli* (**b**) revealed by atomic force microscopy (AFM).

The morphologies of bacterial cells could be visualized using atomic force microscopy (AFM) [52]. Figure 5 shows the height and section images of fresh and treated *E. coli*, the curves presented in the height image correspond to the lines in the section images. For fresh *E. coli* (Figure 5a), the cell had an elliptical shape with a height difference of around 200 nm; meanwhile the cell membrane appeared to be smooth and integrated, implying that the cell remained intact. For the *E. coli* treated with GCS (see Figure 5b), the cells could no longer maintain the elliptical shape. The intracellular contents leaked out from the cell membrane, resulting in the height of bacteria decreasing to 100–150 nm in the section image. The disintegrated cell membrane enables more cytoplasm to diffuse into bacterial suspension, resulting in a high OD_260_ ratio (see Figure 4). Comparing the images of the fresh and treated *E. coli*, it could be concluded that the antimicrobial mechanism of GCS is based on destroying the cell membrane and causing leakage of the intracellular components of *E. coli*.

### 3.4. Adsorption of GCS on the Cellulose Fibers and Hygiene Paper

**Figure 6:**
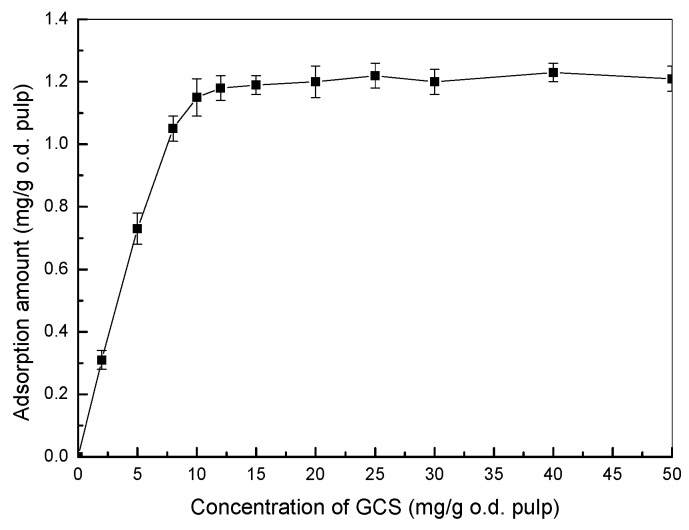
Adsorption of GCS on cellulose fibers (*DS* of GCS = 25.5%).

The obtained GCS is a kind of cationic polyelectrolyte (charge density = 5.28 meq·g^−1^ for GCS with 25.5% *DS*) which is feasible to be adsorbed onto anionic cellulose fibers and used as an antimicrobial additive for hygiene papers. The adsorptions of GCS on fibers are shown in Figure 6. It can be found that the adsorption amount of GCS was increased significantly when the addition concentrations were lower than 12 mg·g^−1^ which indicates a high affinity of the cationic GCS towards cellulose fibers. The adsorption amount leveled off at 1.2 mg·g^−1^ after an addition amount of 12 mg·g^−1^ which means that the increase of GCS concentration does not lead to a corresponding change of adsorption amount after equilibrium.

**Figure 7:**
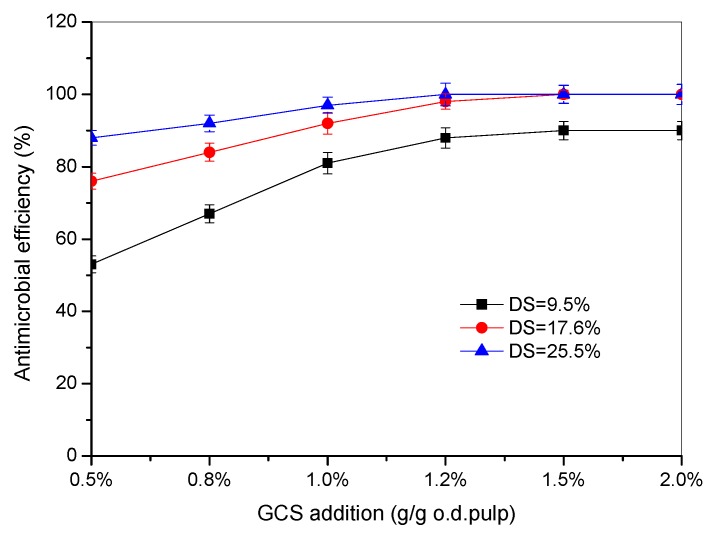
Effects of addition amount of GCS on the antimicrobial efficiency (equilibrium adsorption).

**Figure 8:**
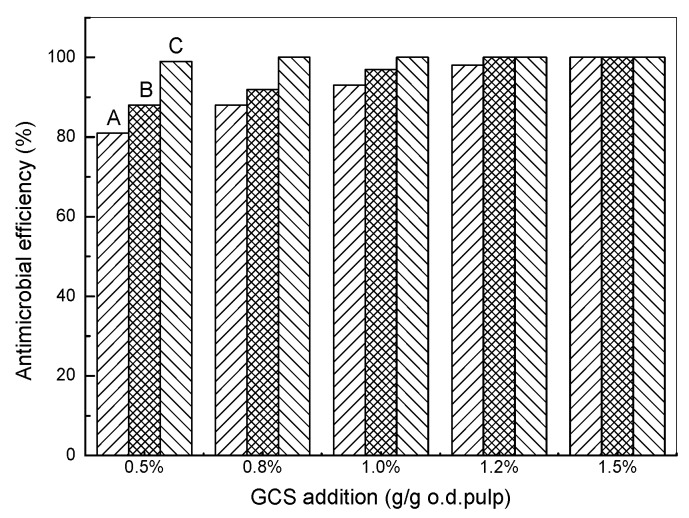
Effects of addition methods on the antimicrobial efficiency (*DS* = 25.5%; (A) non-equilibrium adsorption; (B) equilibrium adsorption; (C) spraying).

The effects of the addition amount of GCS with various *DS* on the antimicrobial efficiency are shown in Figure 7. In general, the antimicrobial efficiency was increased by adding more GCS and it reached a plateau at a certain addition amount. The higher *DS* of GCS is more beneficial for the antimicrobial activity of hygiene papers which is consistent with the results of the dynamic UV absorption. It can be seen that most of the bacteria could be deactivated at an addition level of 1.2% (based on o.d. pulp) for GCS with 25.5% *DS* where it also reached equilibrium adsorption at the concentration. The antimicrobial efficiency of GCS with 17.6% *DS* also reached 100% when the addition amounts were more than 1.5% (based on o.d. pulp). However, the GCS with low *DS* (9.5%) could only deactivate 90% bacteria even when 2.0% (based on o.d. pulp) of GCS was added to the handsheets. Meanwhile, the addition methods of GCS to the handsheets also influenced the antimicrobial efficiency significantly. It is shown in Figure 8 that spraying was most effective to make hygiene papers because the antimicrobial efficiency was nearly 100% when the addition amount of GCS was as low as 0.5% (based on o.d. pulp). Nearly all of the GCS can be retained on the surface of the handsheets by spraying, whereas only a small portion of GCS remains by adsorption onto the cellulose fibers. The adsorption amount of GCS on fibers can be increased to the equilibrium by prolonging the time and should be less for non-equilibrium adsorption with the shorter time, therefore, the antimicrobial efficiency with the non-equilibrium adsorption is lower than that with the equilibrium adsorption.

## 4. Conclusions

A particularly facile approach to prepare guanidinylated chitosan was successfully developed via grafting the guanidine oligomers onto chitosan under microwave irradiation. FT-IR and ^1^H NMR verified the structure of the resultant polymer of covalently bonded guanidine groups to chitosan. The degree of substitution of GCS of 25.5% could be achieved when the molar ratio of the functional groups (PHGC/chitosan) was 2.5:1 at 90 °C and 30 min. The antimicrobial activity of chitosan was highly enhanced by the grafted guanidine oligomers and GCS could inhibit the bacteria rapidly. UV absorption of bacteria suspensions and AFM images of *E. coli* revealed that deactivation of *E. coli* induced by GCS is attributed to the destruction of the cell membrane and release of cytoplasm from the bacteria cells. The equilibrium adsorption of cationic GCS (*DS* = 25.5%) was 1.2 mg·g^−1^ when the addition amount was higher than 12 mg·g^−1^ o.d. pulp, where most of the bacteria could be deactivated. The antimicrobial efficiency of hygiene papers with GCS was influenced by the *DS* of GCS and the addition methods which are relative to the valid guanidine groups retained in the handsheets. This feasible method allows the preparation of chitosan for enhanced antimicrobial activity without the use of tedious processes and broadens its potential application as a biocompatible antimicrobial agent.
